# Importance and usefulness of evaluating self-esteem in children

**DOI:** 10.1186/1751-0759-6-9

**Published:** 2012-03-20

**Authors:** Mizuho Hosogi, Ayumi Okada, Chikako Fujii, Keizou Noguchi, Kumi Watanabe

**Affiliations:** 1 Hosogi Children's Clinic, 2-11-1 Minato-machi, Fukuyama-shi, Hiroshima-ken 721-0964, Japan; 2 Depertment of Pediatirics, Fukuyama Medical Center, 4-14-17, Okinogami-cho, Fukuyama-shi, Hiroshima-ken 721-8520, Japan; 3 Department of Pediatrics, Okayama University Hospital, 2-5-1, Shikata-cho, Kita-ku, Okayama-shi, Okayama-ken 700-8558, Japan; 4 Faculty of Health and Welfare Science, Okayama Prefectural University, 111, Kuboki, Souja-shi, Okayama-ken 719-1197, Japan

**Keywords:** Self-esteem, Psychosomatic disorder, Pope's 5-Scale Test of Self-Esteem for Children, Quality of life, Mental health

## Abstract

Self-esteem is the "feeling of self-appreciation" and is an indispensable emotion for people to adapt to society and live their lives. For children, in particular, the environment in which they are raised contributes profoundly to the development of their self-esteem, which in turn helps them to adapt better to society. Various psychologists have provided definitions of self-esteem, and examined methods of objectively evaluating self-esteem. Questionnaire-style assessment methods for adult include Rosenberg Self-Esteem Scale and Janis-Field Feeling of Inadequacy Scale, and these for children include Coopersmith Self-Esteem Inventory, Pope's 5-Scale Test of Self-Esteem for children, and Kid- KINDL^®^. Other methods include Ziller Social Self-Esteem Scale and Implicit Association Test. The development of children's self-esteem is heavily influenced by their environment, that is, their homes, neighborhoods, and schools. Children with damaged self-esteem are at risk of developing psychological and social problems, which hinders recovery from low self-esteem. Thus, to recover low self-esteem, it is important for children to accumulate a series of successful experiences to create a positive concept of self. Evaluating children's self-esteem can be an effective method for understanding their past and present circumstances, and useful to treat for children with psychosomatic disorders.

## Introduction

UNICEF's adoption of the document "A World Fit for Children" (2002) states that children, including adolescents, must be empowered to exercise their right to expression in accordance with their evolving capacity; build self-esteem; and acquire knowledge and skills needed for conflict resolution, decision-making, communication, and endurance of life's challenges. The World Health Organization's "Preventing Suicide: A Resource for Teachers and Other School Staff" (2000) states that positive self-esteem protects children and adolescents from mental distress and despondency, and enables them to cope adequately with difficult and stressful life situations.

While no consistent views on the definition of self-esteem, how it develops, and its relationship with social adjustment have been established, its importance, particularly for children, has been mentioned at several occasions and is widely accepted as common knowledge. This paper reports on previous definitions, evaluation methods, and ideas for the development of self-esteem, as well as introduces our own research and examines the effectiveness of evaluating self-esteem.

## Definition of self-esteem

Kant and others have argued conventionally from a philosophical and ethical standpoint that self-esteem is "the awareness of the absolute value of one's own personality or dignity." In 1980, James [1] stated that self-esteem is "the satisfaction or dissatisfaction with oneself." In reality, humans select a certain pretension and think of failure in that pretension as a true defeat and of success as a true victory. Feelings such as shame or joy occur as a result, respectively. As such, James saw self-esteem as a ratio found by dividing one's successes in areas of life of importance to a given individual by the failures in them or one's "success/pretensions".

Subsequently, in the field of psychology, self-esteem began to be viewed as "a feeling of self-appreciation." Numerous psychologists have defined and debated the definition of self-esteem, but no uniform view has been established. Various psychologists have provided definitions of self-esteem, and examined methods of objectively evaluating self-esteem.

## Method for evaluating self-esteem

A variety of methods are used for evaluating self-esteem. Examining criteria used is important when interpreting the results of research on self-esteem.

One common measurement method is the use of questionnaires. For adults, examples include Rosenberg Self-Esteem Scale and Janis-Field Feeling of Inadequacy Scale; however, the questions asked in these scales are generally abstract and present difficulties when used for young children. Conversely, measurement methods developed for younger children include Coopersmith Self-Esteem Inventory, Pope's 5-Scale Test of Self-Esteem for children, and Kid- KINDL^®^.

Other methods include projection methods such as Thematic Apperception Test (TAT), in which respondents are asked to create a simple story on the basis of illustrations in TAT, which are then analyzed to reveal a projection of the respondent's concept of "self." Ziller Social Self-Esteem Scale is another type of projection method. In recent years, studies on latent self-esteem that are measured by the computer-driven Implicit Association Test have gained attention. The aforementioned methods are not only used for testing adults but also older children.

### Rosenberg self-esteem scale

Rosenberg [2] was the first to incorporate questionnaires into research on self-esteem. According to Rosenberg, self-esteem is exhibited when "a special object (oneself) has a positive or negative attitude that has basically the same qualities and attitudes toward other objects (other than oneself)." As such, he believed that since attitudes toward objects are measurable, attitudes toward oneself can also be measured. Rosenberg's self-esteem scale comprises questions about 10 different items, and evaluations are made using a four-level scale.

### Janis-field feeling of inadequacy scale

Janis and Field [3] defined self-esteem as "a person's feelings of social adequacy." This scale consisted of a person's feelings of social adequacy: anxiety in social situation, self-consciousness and feelings of personal worthlessness. Janis-Field Feeling of Inadequacy Scale comprises questions about 23 different items, and evaluations are made using a five-level scale.

### Coopersmith self-esteem inventory

Coopersmith [4] defined self-esteem as "positive and negative attitudes toward oneself." He considered self-esteem an expression of approval or disapproval of oneself, and a measure of the extent to which one believes that he or she is talented, successful, and that his or her life has meaning and value. To elucidate the requisite conditions that contribute to the development of self-esteem, Coopersmith created sets of 58 evaluation criteria for children and 50 criteria for adults. In this test, respondents answered each question with either "like me" or "unlike me."

Coopersmith clarified how healthy self-esteem is created by using the test to identify three main conditions: 1) parental warmth and acceptance; 2) clearly defined and enforced limits; and 3) respect for action within these limits.

### Pope's 5-scale test of self-esteem for children

Pope [5] defined self-esteem as the evaluative feelings one holds for oneself and the sense that one has essential worth, and asserted that self-esteem is evaluated as the difference between the actual self and the ideal self. The actual self is based on objective information that the self perceives about itself, that is, the self-concept. The ideal self is an image of the type of person that the individual wishes to be. Self-esteem is high when the actual self and ideal self are in agreement and low when they are discrepant.

Pope's 5-Scale Test of Self-Esteem for Children consists of 60 questions and evaluates self-esteem on 5 scales: Global Scale, Academic Scale, Body Scale, Family Scale, and Social Scale. The maximum score for each scale is 20 points, and the total score for each scale is used for the evaluation. In addition, Lie Scale was established to evaluate response validity for this test.

### Kid- KINDL^®^

Ravens and Bullinger [6,7] defined quality of life (QOL) as "the subjective perception of physical, mental, social, psychological and functional aspects of well-being and health." They developed Kid-KINDL^® ^as an indicator capable of objectively measuring QOL in children. To measure the QOL of children aged 6 to 18 years and develop their criteria, Ravens and Bullinger considered levels of mental and physical health and acclimation at home and school where they spent the most time of the day. The test comprises 24 questions covering six areas: physical health, emotional well-being, self-esteem, family, friends, and school. The total number of points in all six areas supplies the QOL score, with a higher score indicating a higher QOL. Self-esteem is one of the areas comprising QOL, and can thus be individually evaluated.

### Ziller social self-esteem scale

Ziller [8] believed that self-esteem is "the individual's perception of his worth, which comes about from a context of self-other orientation." He pointed out several problems with previous researches concerning self-esteem, including the facts that 1) the social nature of the self-system was not sufficiently emphasized, 2) they were of a descriptive nature, and 3) they largely involved a verbal self-report measure of self-esteem. He went on to introduce a unique evaluation procedure using topological representations of self and others and involving limited verbal demands. For example, six different items based on "yourself" and five different categories on "significant others" are given; the subject enters each item into one of the six horizontally-arranged circles. Results showed that social objects with greater value tend to be placed to the left in the horizontal display; the absolute difference between location of self and a low-status social object is significantly associated with the left to right location of the self; left-right location of the self is significantly associated with the up-down location of the self.

### Implicit association test

Evaluation methods that use questionnaires have long been criticized as being susceptible to self-delusion, social circumstances, and social norms due to subjects being able to control their responses [9]. Measurements of self-esteem are no exception. Shiomura [10] reports that measures of self-esteem also simultaneously measure self-efficacy. In contrast to "explicit self-esteem," studies are also being conducted on "implicit self-esteem," in which subjects are unaware and cannot consciously control their responses.

Implicit Association Test (IAT), conceived by Greenwald, McGhee, and Schwartz [11], is an examination that evaluates subjects' latent attitudes. As per the computerized test, subjects respond to stimulus words displayed on-screen by pressing keys corresponding to the left and right sides to categorize the words into four different concepts appearing on the left and right sides of the screen. Self-esteem is measured by the speed at which the answer is given (i.e., response time). Further, it considers the strength of the association of a series of positive or negative attributes (pleasant or unpleasant, for example) with oneself within each decision task category. The inability of participants to willfully manipulate their IAT scores has also been verified by the experiment.

## Development of self-esteem

The development of a child's self-esteem is heavily influenced by the environment in which he or she is raised. Harter [12] reported that positive self-esteem creation is based on 4 factors: 1) the parent-child relationship; 2) the means used to cope with the child's undesirable emotions; 3) self-acceptance; and 4) social behavior.

Coopersmith [4] noted that the parent's child-rearing behavior has an influence on their children's self-esteem. In his study, parents of children with low self-esteem were characterized by such factors as low self-esteem and emotional instability; moreover, they created an environment that was impoverished physically, emotionally, and intellectually, showed little concern for the child, and reacted to the child in the extreme. The home is the first place in which children build relationships with other people. The ability to have a positive view of oneself is impacted by the way in which children are treated by their parents.

As children grow, the areas where they build relationships expand to their neighborhood and school. Once they reach the age of schooling, children begin to evaluate themselves on the basis of mutual relationships with teachers and friends from academic, social, emotional, and physical aspects [13]. Achievements and accomplishments in these areas increase children's self-esteem and simultaneously form the basis for its further development.

Other research shows that self-esteem changes as children grow. Using Coopersmith Self-Esteem Inventory, Kokenes [14] conducted an examination of children from grade four to eight, and found that self-esteem is lowest in grade six. The study revealed that the elements causing self-denial are more pronounced and elements causing self-acceptance are reduced for sixth graders. As children develop, differences are also likely to arise in the factors that comprise self-esteem and the importance assigned to each of these factors. Shibata [15] and Matsuzaki [16] conducted a Kid- KINDL^® ^survey of Japanese elementary and junior high school students and found that self-esteem declines as children grow older. Many other similar reports indicate that early adolescents exhibit decreased self-esteem, and thus evaluation during these ages should be conducted cautiously.

There is also a difference between the sexes in self-esteem. Endo [17] reports that girl, in general, have lower self-esteem than boys in the measures used by Rosenberg, Janis and Field, and Coopersmith. Shibata [15] and Matsuzaki [16] reported similar findings in their Kid- KINDL^® ^study.

## Problems that cause lower self-esteem and countermeasures

Healthy self-esteem supports psychological stability and positive social activity and is an essential element for a child's psychological development. Many studies indicate a link between low self-esteem and a variety of psychological problems.

In Japan, the issue of children not being able to attend school first began garnering attention during the last half of the 1950s. Among other things, the Ministry of Education, Culture, Sports, Science, and Technology indicates that "inadequate self-esteem" is one factor causing non-attendance at school. Kasuya [18] reports that among junior high school students, the children with non-attendance at school had lower self-esteem than a control group, and Masuda [19] similarly suggests that adolescent girls who skip school have lower self-esteem than those who do not.

It was indicated that the presence of a psychiatric disorder in adolescents is associated with decreased self-esteem [20]. Mendelson [21] and Eiber [22] observed low self-esteem in adolescents and young adults with eating disorders, while Roberts [23] found that adults with depression similarly had low self-esteem. Gayman [24] noted that low self-esteem was linked to the history and timing of depression in young adults. A possible explanation is that these patients were unable to develop adequate self-esteem as children, which prevented them from adopting an effective approach for dealing with stress, leading to the onset of mental disorders.

In addition, there are also many reports of chronic physical illnesses contributing to a decline in children's self-esteem. Studies conducted on social maladaptation and QOL of children with obesity [25], heart disease [26], or chronic kidney disease [27] revealed that the development process of self-esteem suffers from maladaption caused by physical disorders, and as a result, positive social activities are limited and social maladaptation gets worse.

Recently many studies have noted that the prognosis of children with some diseases or problems was determined by the resilience of the child, which is made by resource of circumstance, good relationships with other people, and self-esteem. It is important for children to recover lost self-esteem in their treatment. Accumulating a series of successful experiences can help strengthen their concept of self as something positive. They are likely to have a positive concept of self if they feel they are able to adequately fulfill their role, depending on the group they belong to (e.g., family, class). At school, it is important for teachers to provide children with many opportunities for academic achievement and to interpret their experiences in a positive light.

## Usefulness of evaluating self-esteem in children with psychosomatic disorders

In treating children with psychosomatic disorders, we noted that intractable patients share common characteristics, such as low self-evaluation and complaints of feeling disrespected or unwanted at school or home. We concluded that an evaluation axis, different from that used for diagnosis, is needed to understand the level of pathology and estimate the prognosis in such cases, and that evaluating self-esteem would be useful for this purpose. We attempted to quantify self-esteem in children with psychosomatic symptoms using Pope's 5-Scale Test of Self-Esteem for Children [5], and examined the characteristics and prognosis of patients with low self-esteem [28]. We review a part of this study below.

Although this study showed that low self-esteem across several different areas affects patient prognosis, results also indicated that low self-esteem in one area alone does not necessarily have a negative impact. Resnick [29] noted that family and school contexts as well as individual characteristics are associated with psychological problems in adolescents. Our examinations led us to conclude that psychological problems are more likely to occur in children if there are problems in at least two of these three elements. Similarly, if self-esteem has been damaged in several different areas, then maladjustment to a degree that requires medical consultation is likely to develop and treatment is likely to be difficult. These results imply, however, that even children with many different issues can show improvement without necessarily improving self-esteem in all areas of life, that is, if their self-evaluations are augmented by acceptance by someone at home, school, or elsewhere.

Furthermore we are examining self-esteem in children with non-attendance at school using Pope's 5-Scale Test of Self-Esteem for Children. In this study self-esteem is compared between the children with non-attendance at school and those who attend school. Therapists and parents tend to think that self-esteem in children with non-attendance at school is low in Social Scale. However, in actually their self-esteem is significantly low in Academic Scale and Family Scale. We can support both areas with comparative ease using methods such as individual study guidance and change of relationships between family members. Evaluating self-esteem facilitate the establishment of tangible treatment plans.

Our institution uses Pope's 5-Scale Test of Self-Esteem for Children as part of the examinations and uses the same in our treatment. A case study is provided below.

Case study: 14-year-old male

Diagnosis: Irritable Bowel Syndrome, Generalized Anxiety Disorder

Process: The subject suffered from abdominal aches in June of the first year of middle school. His abdominal issues worsened over time and symptoms of anticipatory anxiety developed. Thus, the patient was prohibited from leaving the house. He visited our hospital in March of the following year with complaints of abdominal pains leading to school absence. He was diagnosed with irritable bowel syndrome and began medicinal treatment. Physical symptoms improved but the patient was afraid to go outside, and thus was unable to return to school. He began inpatient treatment in May of the third year of middle school. The patient completed Pope's 5-Scale Test of Self-Esteem for Children upon admission (Table 1). After admission, he began interacting with others within the in-hospital classes and gained confidence in relationships with others. Upon reflection of his relationship with his parents, we were able to reaffirm that he was accepted within his family. He completed Pope's 5-Scale Test of Self-Esteem for Children again upon discharge, and showed improvement in areas other than Academic Scale and Body Scale. Conversely, because scores in Academic Scale continued to be low, the school was asked to focus on providing remedial study after he returned to school. Individual study guidance was given, and the patient graduated to high school. Medical problems stabilized and treatment was ended.

**Table 1:** The change of self-esteem

	On Admission	At time of Discharge
Global Scale	3	10

Academic Scale	4	3

Body Scale	4	5

Family Scale	8	15

Social Scale	8	15

## Discussion

If we assume that low self-esteem has an impact on patient prognosis, then it is beneficial from a therapeutic standpoint to identify and address the causes of low self-esteem. Our study revealed that family dysfunction problems such as a family member with a psychiatric disorder, economic hardship, or experience of child abuse have an impact on self-esteem. Moreover, patients and their families often do not report such problems to their therapists. As such, this evidence gives reason to suspect that certain problems may exist within the home of seemingly normal families. Thus, when therapists discover that a child has low self-esteem in many different areas, an assessment of family functionality is necessary.

Therapists should consider children with such family dysfunction problems as being at risk of having low self-esteem and should take appropriate action at an early stage. Further, increased support from outside the family can be effective when encouraging the child, particularly when the support from his or her family is insufficient. Self-esteem can be improved if schools, juvenile consultation centers, and others work together to create a haven outside of the home where children feel accepted. In such cases, treatment should first be approached from areas that facilitate cooperation, for example, public health nurses and school nurses can supplement the role of parents by providing encouragement and information to children in regard to their future.

We conclude that a non-diagnostic evaluation axis is necessary to understand the pathological condition of children with psychosomatic disorders and project the treatment prognosis and that assessment of self-esteem can be an effective method.

## Conclusion

Self-esteem is the "feeling of self-appreciation" and is an indispensable emotion for people to adapt to society and live their lives. For children, in particular, the environment in which they are raised contributes profoundly to the development of their self-esteem, which in turn helps them to adapt better to society. Children with damaged self-esteem are at risk of developing psychological and social problems, which hinders recovery from low self-esteem. Evaluating children's self-esteem can be an effective method for understanding their past and present circumstances, and useful to treat for children with psychosomatic disorders.

## Competing interests

The authors declare that they have no competing interests.

## Authors' contributions

The authors wrote the manuscript and holds final responsibility for the decision to submit the manuscript for publication.
